# Mechanism Study on the Preventive Effect of ELITEA Compound Tea on Hyperuricemia in Rats Based on Serum Untargeted Metabolomics

**DOI:** 10.3390/metabo15050336

**Published:** 2025-05-19

**Authors:** Shulian Liu, Yongliang Zhu, Wenjiong Wang, Xianghan Zhang, Linrui Gao, Xiangjun Qiu

**Affiliations:** 1 School of Nursing and Health Care, Luoyang Polytechnic, Luoyang 471000, China; liushulian@lypt.edu.cn (S.L.); zylsy@lypt.edu.cn (Y.Z.); 2 College of Basic Medicine and Forensic Medicine, Henan University of Science and Technology, Luoyang 471023, China; wangwenjiong@stu.haust.edu.cn (W.W.); zhangxianghan@stu.haust.edu.cn (X.Z.); 3 School of Tea and Coffee, Pu’er University, Pu’er 665000, China; gaolinrui@peu.edu.cn

**Keywords:** hyperuricemia, metabolomics, ELITEA compound tea, Pu’er tea

## Abstract

**Background/Objectives**: Hyperuricemia (HUA) has become the second largest metabolic disease after diabetes, and has become a major public health problem. The ELITEA compound tea extract can effectively reduce the serum uric acid level in HUA rat models. In this study, the mechanism of ELITEA compound tea on HUA was analyzed through serum untargeted metabolomics analysis. **Methods**: The rat model of HUA was established by feeding rats with a high uric acid diet. A total of 24 male SD rats were divided into a blank control group, a hyperuricemia model group, and an ELITEA compound tea prevention experimental group. UPLC-MS/MS was used to detect changes in metabolites in the blood of the three groups of rats, in order to identify potential biomarkers and study the mechanism of ELITEA compound tea in preventing HUA. **Results**: The ELITEA compound tea exhibited significant preventive effects on HUA rats. The analysis results showed that after ELITEA combined tea intervention, the 257 metabolites downregulated in the HUA model group showed an upward trend. Meanwhile, the 115 metabolites upregulated in the HUA model group showed a decreasing trend. Six main enrichment pathways were obtained, including arginine biosynthesis, histidine metabolism, pyrimidine metabolism, tryptophan metabolism, vitamin B6 metabolism, arginine and proline metabolism. **Conclusions**: ELITEA compound tea can effectively reduce the serum uric acid levels in HUA model rats. Based on the in-depth analysis of untargeted metabolomics, ELITEA compound tea mainly regulates the arginine biosynthesis pathway by modulating three important metabolites, arginine, glutamate, and ornithine, to reduce serum uric acid.

## 1. Introduction

Uric acid, as the end metabolite of purine metabolism, plays a pivotal role in reflecting the health status of an individual. It is estimated that roughly sixty to seventy percent of uric acid is eliminated from the body via the urinary system, with the remaining thirty to forty percent being discharged through other excretory processes [1]. Hyperuricemia (HUA) is a common and potentially destructive chronic metabolic disease caused by the accumulation of urate salts in the body due to excessive uric acid production or reduced excretion caused by purine metabolism disorders in the body. The human body itself lacks uricase, and with the continuous development of the social economy, human lifestyle has undergone significant changes. The occurrence of HUA is on the rise annually, and it is progressively affecting individuals at a younger age [2]. HUA has become the second largest metabolic disease after diabetes, and has become a major public health problem [3]. And with the increasing prevalence of HUA year by year, more and more studies have found that HUA is related to cardiovascular disease, hypertension, diabetes, dyslipidemia and other metabolic related diseases.

HUA often has no obvious clinical symptoms, so it is easily overlooked. Therefore, preventing the occurrence of HUA is of great significance to human health [4]. Although the use of uric acid-lowering drugs can rapidly reduce uric acid levels, it is often accompanied by the occurrence of some adverse reactions. By contrast, reducing uric acid levels through dietary adjustments is considered a milder and more sustainable approach. This method not only reduces the side effects caused by medication, but also helps improve overall health. A higher dietary diversity score may reduce the risk of hyperuricemia incidence compared to a lower dietary diversity score [5]. A nutritionally balanced diet, i.e., consuming more grains and fresh fruits and vegetables, as well as quitting smoking and reducing alcohol consumption, has been proven to effectively prevent the occurrence of HUA [6]. Chinese sumac fruit extract can improve HUA and uric acid nephropathy in mice fed a high-purine yeast diet. This finding establishes a theoretical foundation for developing Chinese sumac fruit as a functional food or medicine for preventing and treating HUA [7]. Properly increasing water intake, especially before bedtime, is beneficial for the excretion of nocturnal metabolites through the kidneys [8]. Drinking an insufficient amount of water daily is associated with increased arterial stiffness and has a negative effect on vascular health in metabolic women with hyperuricemia [9]. In addition, studies have found that drinking tea can also reduce the risk of developing HUA [10]. Drinking tea may lower uric acid levels in male steelworkers and steelworkers who prefer smoked and pickled foods [11].

Tea is considered to be one of the most important contributions of the Chinese nation to human civilization. Yunnan Province in China is considered to be the origin of the world’s tea trees. Pu’er tea is produced in Yunnan Province, which is protected by geographical indications. It uses Yunnan Province’s unique large leaf sun-dried tea leaves as raw materials and is carefully crafted through a series of specific processing techniques carried out within geographical indication protection areas. The Dayi ELITEA compound tea produced by Yunnan Dayi Microbial Technology Co., Ltd (Menghai China). draws on the essence of Xishuangbanna Pu’er tea. This tea adopts advanced third-generation intelligent fermentation technology with the goal of promoting health, namely the microbial tea-making process, providing consumers with a brand-new tea experience. The fermented Pu’er tea produced through the microbial tea-making method has more beneficial ingredients and a higher nutritional value, and therefore represents a milestone in the history of Pu’er tea development. Our previous research results have shown that the ELITEA compound tea extract can effectively reduce the serum uric acid level in HUA rat models, reduce damage to the liver and kidneys, and have a certain preventive effect and positive impact on HUA [12]. The protective mechanism of TAETEA Prebiotea on non-alcoholic fatty liver was studied through non-targeted metabolomics [13].

Metabolomics is a scientific field that focuses on analyzing all small molecule metabolites within an organism, typically with a relative molecular weight not exceeding 1000. It mainly uses chromatography–mass spectrometry and nuclear magnetic resonance techniques to identify and quantify these small molecules. The key to this discipline lies in the high sensitivity and stability of its analytical methods, as well as its deep dependence on databases, which together ensure the precise capture of metabolic changes within living organisms. Metabolomics has a wide range of applications, especially in disease diagnosis. Due to the differences in metabolites under different disease states, metabolomics can reveal these specific changes, providing new biomarkers for early disease identification and the differentiation of disease states. By analyzing the patterns and changes of these metabolites, scientists can better understand the biological basis of diseases and develop new diagnostic tools and treatment methods [14]. This can also allow for the evaluation of disease diagnosis and prognosis, treatment evaluation, and toxicology research [15]. Metabolomics is a new technology. In recent years, it has been successfully applied in many fields, from its initial use in discovering new mechanisms and biomarkers to its application in theoretical research in food and nutrition [16,17].

This study established a HUA rat model and measured serum uric acid levels in the plasma of the blank control group, HUA model group, and ELITEA compound tea prevention experimental group to clarify the preventive effect of ELITEA compound tea. Untargeted metabolomics analysis was conducted utilizing an ultra-high performance liquid chromatography system coupled with a four-stage rod orbital trap mass spectrometer. Combined with the overall metabolite changes of the samples, the metabolic changes of the rat HUA model group and the ELITEA compound tea prevention experimental group were studied, providing a metabolomics basis for studying the preventive mechanism of ELITEA compound tea on HUA disease. Through metabolomics analysis, the mechanism of ELITEA compound tea in preventing HUA was predicted, and the reference for the daily prevention of HUA was provided.

## 2. Materials and Methods

### 2.1. Instrument, Reagent, and Tools

The iMagic-7 fully automatic biochemical analyzer was from Shenzhen Kubel. The Nexera X2 LC-30AD ultra-high performance liquid chromatography system (UHPLC) was from Shimadzu (Tokyo, Japan), and the ACQUITY UPLC HSS T3 column (2.1 × 100 mm, 1.8 µm) was from Waters (Milford, MA, USA). The QE Plus mass spectrometer was from Thermo Scientific (Waltham, MA, USA). The Concentrator Plus vacuum centrifuge and Centrifuge 5424R centrifuge were both from Eppendorf (Hamburg, Germany).

The ELITEA compound tea (YiYuanSu, U formula, 20230115) was produced by Yunnan Dayi Microbial Technology Co., Ltd. (Menghai, China). HPLC-grade formic acid, methanol, and acetonitrile were all purchased from Millipore (Bedford, MA, USA). Ammonia was purchased from Merck (Darmstadt, Germany). The specialized feed for modeling hyperuricemia rats was purchased from Huaxing Experimental Animal Farm in Huiji District, Zhengzhou City (Zhengzhou, China), while the maintenance feed for ordinary rats was purchased from Beijing Keao Cooperative Feed Company (Beijing, China).

The analysis software used was as follows: Progenesis QI v3.0 software (Nonlinear Dynamics, Newcastle, UK), MS-DIAL (ver. 4.70, University of California, Davis, CA, USA and Riken Institute, Japan), Python software (3.10, Python Software Foundation, NL), SIMCA-P 14.1 (Umetrics, Umea, Sweden), Venny (ver. 2.1.0, https://bioinfogp.cnb.csic.es/tools/venny/index.html (accessed on 20 October 2023)).

### 2.2. Experimental Animals

A group of 24 robust male Sprague Dawley (SD) rats, with body weights ranging from 180 g to 220 g, were carefully selected. These rats with the production license number SCXK (Henan) 2019-0002 were purchased from Huaxing Experimental Animal Farm in Huiji District, Zhengzhou City. The rats were fed with adaptive feeding for one week in a standard experimental cage. The temperature of the rat feeding environment was controlled at 22 ± 2 °C, the humidity was controlled at 55 ± 5%, the light–dark cycle was 12 h, and normal diet and drinking water were ensured. The animal experimental plan had been approved by the Animal Laboratory of Henan University of Science and Technology, approval number 202307003. Throughout the entire experimental process, animal experiments followed the Guide for Ethical Review of Laboratory Animal Welfare (GB/T35892-2018) [18].

### 2.3. Experimental Steps

A total of 24 SD rats were assigned into three distinct cohorts: a blank control group, a HUA model group, and an ELITEA compound tea prevention experimental group. The blank control group had unrestricted access to standard rodent chow. The HUA model group and ELITEA compound tea prevention experimental group were both freely fed with HUA rat modeling specific feed, and each group drank normal water. During the feeding process, the ELITEA compound tea prevention experimental group rats were given 200 mg/mL (5 mL/kg) of ELITEA compound tea solution, while the blank control group and HUA model group rats were given 5 mL/kg of physiological saline solution, orally administered twice a day for 28 days.

On the 29th day of the experiment, all rats were anesthetized by an intraperitoneal injection of 3% pentobarbital sodium at a dose of 30 milligrams per kilogram of body weight after 12 h of fasting and water deprivation. Subsequently, a vacuum blood collection tube was used for abdominal aortic puncture to collect blood samples. The collected blood samples were treated with heparin anticoagulation and centrifuged at a speed of 3000 revolutions per minute for 15 min. Finally, serum was extracted and stored at −80 °C.

### 2.4. Blood Uric Acid Concentration Detection Method

After completely thawing the serum samples stored at −80 °C, they were placed in a fully automated biochemical analyzer to detect the blood uric acid concentration values of each group of rats. The blood uric acid concentration data of each group was presented in the form of mean ± standard deviation (Mean ± SD), and statistical analysis was performed using a *t*-test.

### 2.5. Untargeted Metabolomic Experimental Methods

#### 2.5.1. Extraction of Metabolites and Preparation of QC Samples

Firstly, we placed the serum sample stored at a low temperature in a 4 °C environment for gentle thawing. After thawing, we used a vortex mixer to thoroughly mix the sample to ensure the even distribution of ingredients. Next, we accurately transferred 100 μL of serum sample to an EP tube and added 400 μL of pure methanol solution pre-cooled to 4 °C. After mixing again, we placed the EP tube in an ice bath and sonicated it for 20 min to promote solvent penetration and sample dissolution. After ultrasonic treatment, we placed the EP tube at −20 °C for 1 h to allow protein and other macromolecules to precipitate. Afterwards, it was centrifuged at 4 °C for 20 min using a centrifugal force of 16,000× *g* to separate the precipitate and the supernatant. After centrifugation, we carefully collected the supernatant and used a high-speed vacuum concentration centrifuge to completely dry it, preparing it for subsequent mass spectrometry detection.

In the mass spectrometry detection stage, we added 100 μL of methanol–water mixed solution (volume ratio 1:1) to the dried sample to resuspend the sample. This was centrifuged again with 20,000× *g* of centrifugal force at 4 °C for 15 min to remove any possible small particles or precipitates. After centrifugation, we took the upper clear liquid as the test sample for mass spectrometry analysis.

In this experiment, in order to ensure the stability of the chromatography–mass spectrometry system and accurately evaluate the instrument status, we specially prepared quality control (QC) samples. These QC samples were prepared by mixing all the test samples in equal quantities, with the aim of continuously monitoring the consistency and repeatability of the system throughout the entire experimental process. A total of four QC needles were inserted, with the insertion rule being as follows: one QC needle at the beginning, one QC needle after nine samples, and finally inserting two QC needles.

#### 2.5.2. Chromatographic Separation

During the analysis process, the samples were properly stored in an automatic sampler at 4 °C. The injection volume was set to a precise 4 μL and the column temperature was maintained at 40 °C, providing ideal temperature conditions for chromatographic separation. The flow rate of the mobile phase was precisely controlled at 0.3 mL/min. The chromatographic system used a 0.1% formic acid aqueous solution as the mobile phase A, which helps to improve the separation efficiency of certain compounds in an acidic environment. Meanwhile, acetonitrile was used as mobile phase B, which has low polarity, and helps to achieve the effective separation of different compounds during the chromatographic process. An isocratic elution was first performed using 0% Buffer B for 2 min. Subsequently, the proportion of Buffer B was linearly increased to 48% over the next 4 min. Thereafter, the proportion of Buffer B was continually increased to 100% within 4 min, and then the proportion was maintained for 2 min. Upon the completion of the elution, the proportion of Buffer B was rapidly reduced to 0% within 0.1 min, i.e., using exclusively Buffer A. Finally, a 3-min re-equilibration period was performed.

#### 2.5.3. Mass Spectrometry Collection

Each specimen was identified in both positive and negative ionization modes through the application of electrospray ionization (ESI). The specimens were fractionated using UHPLC and subsequently subjected to mass spectrometry analysis with a heated electrospray ionization (HESI) source for ion generation. The ionization parameters were set as follows: spray voltage at 3.8 kV for the positive mode and 3.2 kV for the negative mode; the capillary temperature was maintained at approximately 320 °C with a minor variance; sheath gas flow was set to 30 with a slight fluctuation; auxiliary gas flow was at 5 with a minor variance; the probe heater temperature was regulated at around 350 °C with a minor variance; and the S-lens radio frequency (RF) level was fixed at 50.

The mass spectrometry data acquisition spanned a duration of 15 min. The mother ion scanning range was set between a 75 to 1050 mass-to-charge ratio (*m*/*z*), with the primary mass spectrometry operating at a resolution of 70,000 at *m*/*z* 200. The automatic gain control (AGC) target was set to 3 × 10^6^, and the maximum ion time (IT) for level 1 was capped at 100 milliseconds.

For the secondary mass spectrometry analysis, the procedure was as follows: post each complete scan, the secondary mass spectrometry spectra (MS2 scan) were activated for the top 10 parent ions with the highest intensity. This was achieved with a resolution of 17,500 at *m*/*z* 200, an AGC target of 1 × 10^5^, a secondary maximum ion time of 50 milliseconds, and the MS2 activation type was high-resolution collision-induced dissociation (HCD). The isolation window was set to *m*/*z* 2, and the normalized collision energy was stepped through levels of 20, 30, and 40.

#### 2.5.4. Data Preprocessing

The raw data was processed by MS-DIAL (ver. 4.70) analysis to complete peak alignment, the correction of the retention time, and the calculation of the peak area. In the metabolite identification stage, we used precise mass number matching techniques to ensure that the mass deviation did not exceed 10 ppm, and further controlled the mass deviation within 0.01 Da through secondary spectrum matching to retrieve public databases such as HMDB, MassBank, and GNPS. After completing the data extraction, we screened out ion peaks with missing values exceeding 50% within the group to ensure the reliability of the statistical analysis. For positive and negative ion data, we normalized the total peak area and integrated the positive and negative ion peaks. Subsequently, pattern recognition analysis was conducted using Python software (3.10). Before conducting data analysis, we performed a preprocessing step of unit variance scaling on the data to improve its analysis efficiency.

## 3. Results

### 3.1. Changes in Serum Uric Acid Levels in Rats

**Figure 1:**
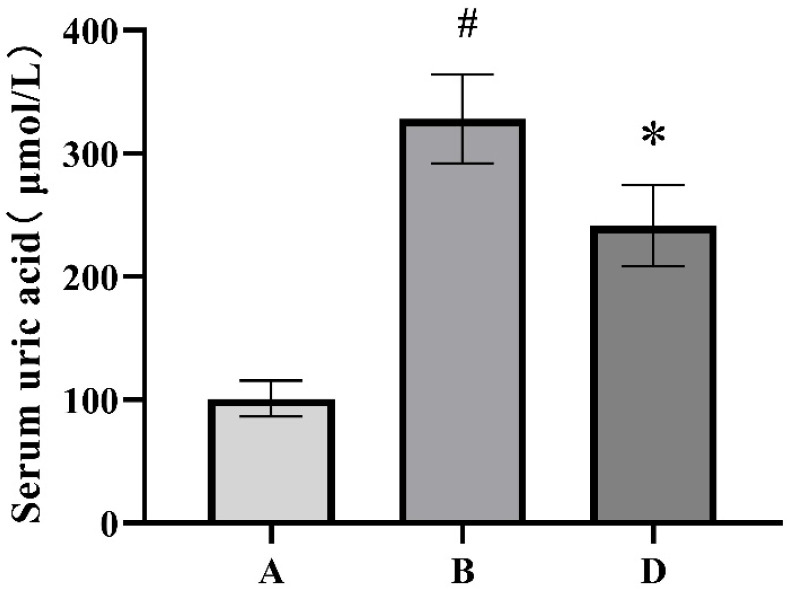
Comparison chart of serum uric acid levels in each group of rats. A: Blank control group; B: Hyperuricemia model group; D: ELITEA compound tea prevention experimental group. * Compared with the A group, *p* < 0.05, # compared with the B group, *p* < 0.05. A: Blank control group; B: HUA model group; D: ELITEA compound tea prevention experimental group.

The serum uric acid levels of each group of rats are shown in Figure 1. Compared with the blank control group, the serum uric acid level in the HUA model group significantly increased, indicating the successful establishment of the HUA rat model. Compared with the HUA model group, the serum uric acid level of the ELITEA compound tea prevention experimental group significantly decreased, indicating that ELITEA compound tea can reduce the serum uric acid level of rats and has a certain preventive effect on HUA.

### 3.2. Results of Untargeted Metabolomics Research

#### 3.2.1. Experimental Quality Evaluation

**Figure 2:**
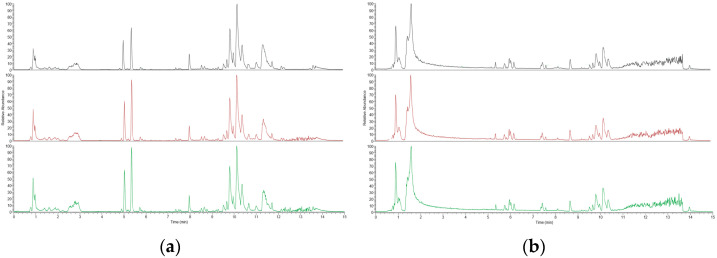
Positive ion mode spectrum of QC samples: (**a**) Positive ion mode spectrum of QC samples; (**b**) Negative ion mode base peak spectrum of QC samples.

In this study, we used a positive and negative ion detection mode to comprehensively display the mass spectrometry total ion chromatogram of the QC samples, which are shown in Figure 2, respectively. Through careful comparative analysis, we observed that the response intensity and retention time of different chromatographic peaks showed high consistency. This discovery strongly confirms that the errors caused by the instrument during the experimental process were very small, ensuring the reliability and accuracy of the data.

**Figure 3:**
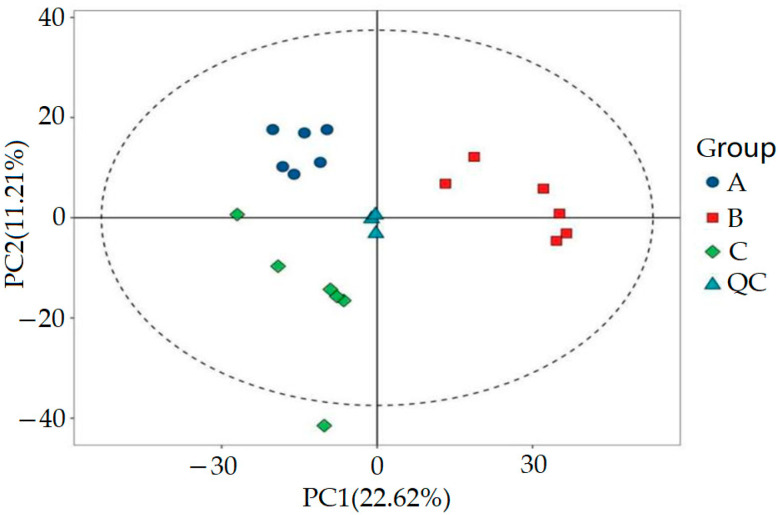
PCA score plot of QC samples.

Furthermore, we utilized MSDIAL software (ver. 4.70, University of California, Davis, USA and Riken Institute, Japan) to accurately extract the metabolite ion peaks. In the positive and negative ion modes, we collected a total of 36,789 ion peaks. In order to conduct in-depth analysis of these data, we extracted peak data from all experimental and QC samples, processed them with UV, and performed principal component analysis (PCA). In the PCA process, data centralization is the primary step. It involves processing the raw data by subtracting the mean of each feature to ensure that the mean of the data is zero. Next, we calculated the covariance matrix of the data, which covers the covariance information between all of the features. Covariance, as an indicator of the relationship between variables, plays a crucial role here. Through seven cycles of interactive verification, we constructed a robust PCA model, and the results are shown in Figure 3. The figure unmistakably illustrates that the QC samples were closely grouped, a pattern that underscores the experiment’s remarkable consistency. This tight clustering was also a testament to the experiment’s well-considered and logical design.

Based on our analysis, it could be said with certainty that the instrumental analysis system used in this experiment demonstrated excellent stability. The collected experimental data were not only stable but also highly reliable. In addition, the metabolic mass spectrometry differences identified during the experiment provided us with an effective tool for revealing biological differences between samples.

#### 3.2.2. Multivariable Statistics

**Figure 4:**
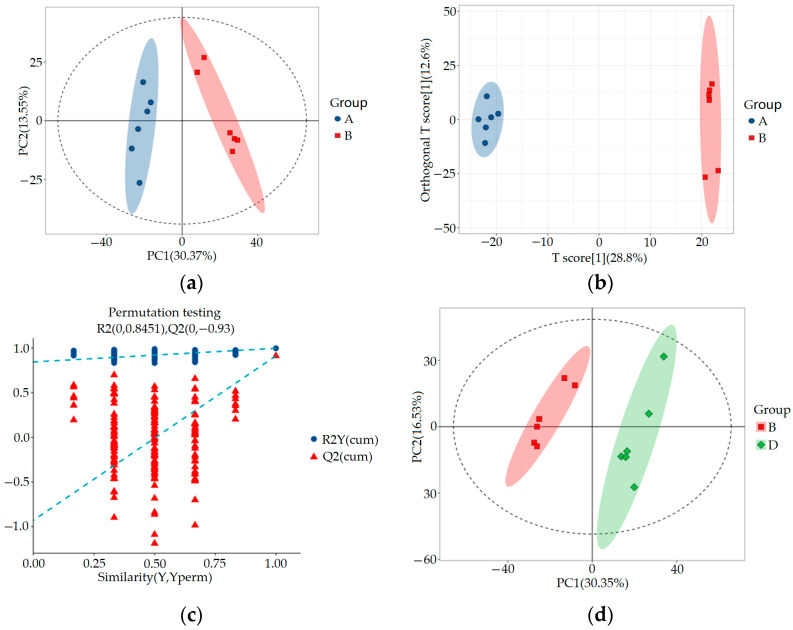

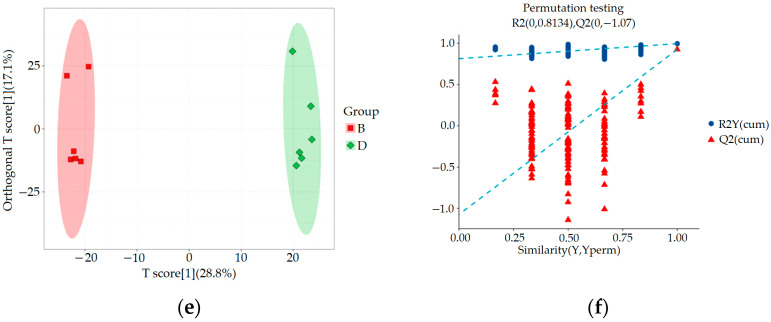
Metabolomics multivariate statistical analysis chart between groups: (**a**) Comparing the PCA scores of groups A and B; (**b**) OPLS-DA score plot for a comparison of groups A and B; (**c**) OPLS-DA permutation test chart for a comparison of groups A and B; (**d**) Comparing the PCA scores of groups B and D; (**e**) OPLS-DA score plot for a comparison of groups B and D; (**f**) OPLS-DA permutation test chart for a comparison of groups B and D.

**Figure 5:**
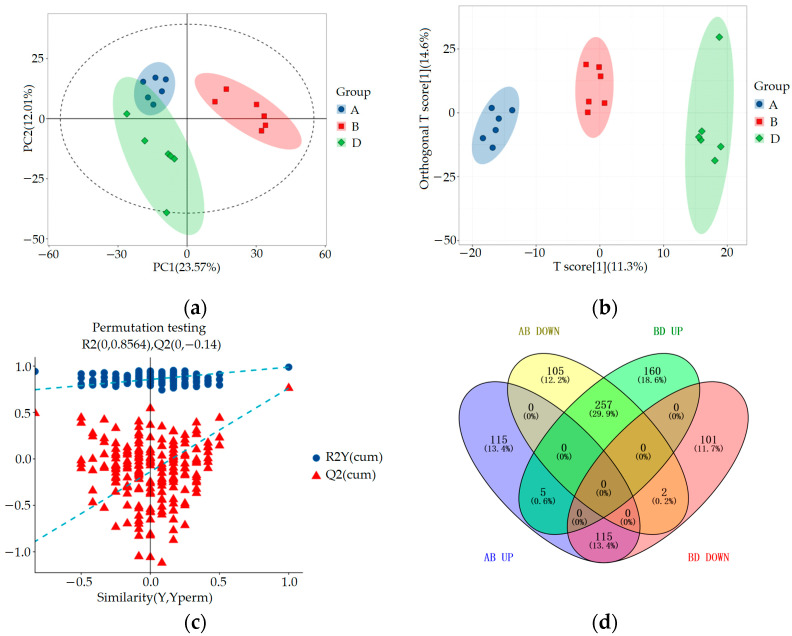
Metabolomics multivariate statistical analysis chart and Venny diagram of differential metabolites among groups A, B, and D: (**a**) Comparing the PCA scores of groups A, B, and D; (**b**) OPLS-DA score plots for a comparison of groups A, B, and D; (**c**) Comparison of OPLS-DA permutation test graphs for groups A, B, and D; (**d**) Venny diagram of differential metabolites among groups A, B, and D.

SIMCA-P 14.1 (Umetrics, Umea, Sweden) was used for PCA, orthogonal partial least squares discriminant analysis (OPLS-DA), and OPLS-DA permutation test analysis to evaluate the accuracy of the model. The results are shown in Figure 4 and Figure 5. By comparing the PCA score plot, OPLS-DA score plot, and OPLS-DA permutation test plot between the blank control group, HUA model group, and ELITEA compound tea prevention experimental group, it can be seen that the samples were closely clustered together. Meanwhile, Table 1 shows that in each comparison group, the intercept of the Q2 regression line in the OPLS-DA permutation test was less than 0, and R2Y&Q2 was greater than 0.5, approaching 1. This indicated that the OPLS-DA model established based on experimental data had not experienced overfitting, and that the model established in this study was very reliable.

#### 3.2.3. Significant Differential Metabolite Analysis

**Figure 6:**
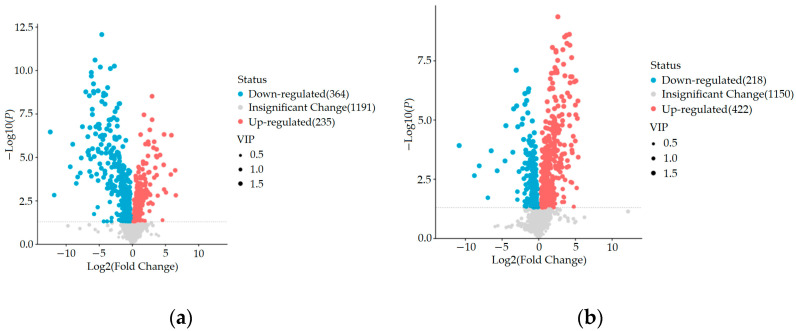
Comparison of volcano maps between different groups: (**a**) Blank control group and HUA model group; (**b**) HUA model group and ELITEA compound tea prevention experimental group.

Through untargeted metabolomics analysis, we identified a total of 1792 metabolites, including 1018 cationic metabolites and 774 anionic metabolites. Using OPLS-DA VIP > 1 combined with *p* < 0.05 for screening, we compared the blank control group and HUA model group using an inter-group comparison method. We found a total of 599 differential metabolites, including 235 upregulated and 364 downregulated metabolites, as shown in Figure 6a. In comparison with the HUA model group, the ELITEA compound tea prevention experimental group revealed the presence of 640 differential metabolites. Among these metabolites, 422 showed a significant upregulation trend, while 218 showed a significant downregulation trend. This phenomenon is directly demonstrated in Figure 6b.

In order to comprehensively analyze these three sets of data, we used the Venny online tool to visualize the differential metabolites. The analysis results showed that after ELITEA compound tea intervention, 257 metabolites in the HUA model group decreased and showed an increasing trend. Meanwhile, 115 metabolites in the HUA model group showed an upward trend and a downward trend after ELITEA compound tea intervention, as shown in Figure 5d. These 354 differential metabolites were considered as key biological factors for ELITEA compound tea preventing HUA. Table 2 respectively provides specific information on the top 20 significantly different metabolites under two trends.

#### 3.2.4. Metabolic Pathway Analysis

**Figure 7:**
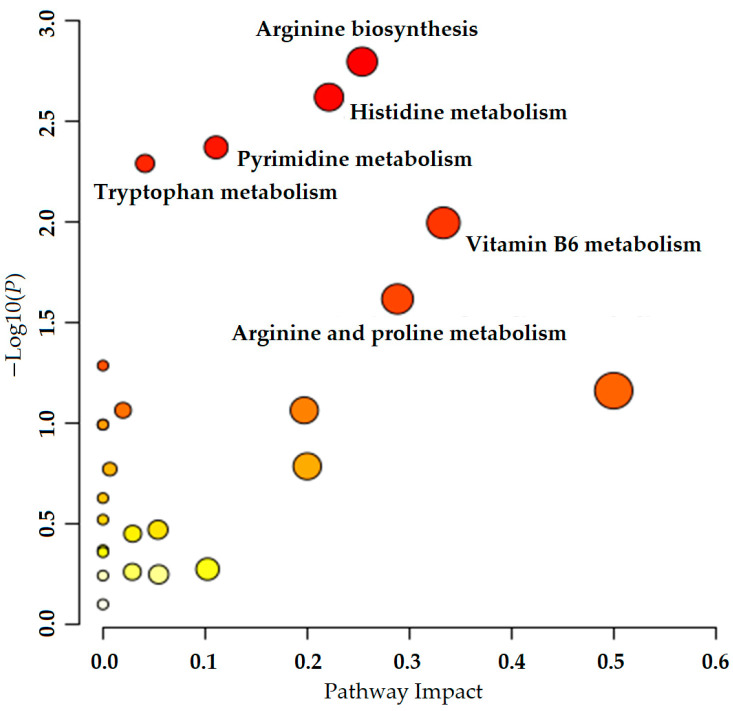
Metabolic pathway analysis of ELITEA compound tea intervention in HUA rats.

The 372 differential metabolites obtained in the significant differential metabolite analysis were identified by HMDB and KEGG databases as relevant differential metabolites that conform to metabolic pathways. These metabolites were imported into the metabolomics analysis platform and analyzed using the pathway analysis function. Screening was conducted under the conditions of *p* < 0.05 and impact >0.1. As a result, six main enrichment pathways were obtained, including arginine biosynthesis, histidine metabolism, pyrimidine metabolism, tryptophan metabolism, vitamin B6 metabolism, and arginine and proline metabolism (see Figure 7). Among these pathways, the *p* value of arginine biosynthesis was the smallest, indicating its significant relationship with ELITEA compound tea and HUA. Therefore, it was considered the most important pathway for ELITEA compound tea to prevent HUA.

## 4. Discussion

HUA is characterized by elevated serum uric acid levels, which are associated with gout, cardiovascular disease, kidney disease, metabolic syndrome, diabetes, and other diseases. The prevalence of hyperuricemia is on the rise globally, especially in most developed countries, which seriously damages the quality of life of those affected [19]. Tea has been widely used in the research of preventing various chronic diseases, and has achieved good relief effects on many diseases, including HUA. Supplementing the tea extract theabrownin (TB) would be a healthy and effective improvement strategy for treating patients with hyperuricemia and intestinal damage caused by environmental factors; TB exhibits significant potential in decreasing serum uric acid levels both in HUA mice and human patients [20,21]. The intake of Pu’er tea promotes the excretion of uric acid and reduces the levels of uric acid and xanthine in serum [22]. The results of this study showed that after administering ELITEA compound tea orally, the plasma uric acid levels in rats were significantly reduced compared to the model group. ELITEA compound tea can effectively reduce serum uric acid levels in HUA model rats [12].

**Figure 8:**
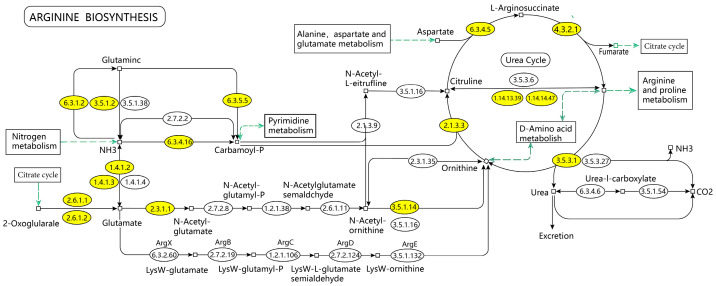
KEGG mapping of arginine biosynthesis.

This study analyzed the mechanism of ELITEA compound tea in preventing HUA through serum untargeted metabolomics, and concluded that the arginine biosynthesis pathway was the most noteworthy metabolic pathway for ELITEA compound tea in preventing HUA. The KEGG diagram of the arginine biosynthesis pathway is shown in Figure 8, which mainly involves six processes: alanine, aspartate, and glutamate metabolism, the citric acid cycle, arginine and proline metabolism, nitrogen metabolism, D-amino acid metabolism, and pyrimidine metabolism. Through further analysis, it was found that the biosynthesis pathway of arginine mainly involves changes in three important metabolites, namely C00062 (arginine), C00025 (glutamic acid), and C00077 (ornithine). These metabolites may play a key role in the pathogenesis of HUA in ELITEA compound tea.

Metabolomics, as a powerful tool, can reveal biomarkers of diseases and delve into the complex relationship between life systems and drugs. It can not only study the changes in the pharmacological components of drugs in vivo, but also explore the targets and metabolic network regulation mechanisms under drug action, thereby revealing the mechanisms of drug effects [23]. In this study, most of the differential metabolites identified were amino acid compounds, and the pathways were mostly related to amino acid metabolism. It can be found that the HUA and ELITEA compound teas are closely related to amino acids. After consuming protein, the human body hydrolyzes it to produce amino acids, which are then broken down and metabolized into other substances. This reaction provides energy for the human body as well as substances for other reactions. If there are abnormalities in the amino acid metabolism pathway, they may lead to the occurrence of various diseases such as HUA.

Arginine is an endogenous synthesized proteinogenic amino acid that can downregulate adenosine deaminase and xanthine oxidase signaling, leading to a halt in uric acid production [24]. Meanwhile, studies have shown a significant correlation between arginine and urinary nitrite, suggesting an increase in endothelial cell NO production. A long-term increase in the dosage of arginine can induce an increase in urinary nitrite excretion [25]. And we can conclude from some studies that [26] the body’s uric acid may strongly bind to arginine residues to promote a decrease in overall uric acid levels.

Glutamate can be taken up by the liver and converted into glutamine, which can produce nucleotides that are ultimately degraded into uric acid, leading to an increase in uric acid levels [27]. Glutamine is also an essential nutrient for maintaining kidney function. Studies have shown that [28] during acidosis, the kidneys increase their intake of glutamine, which then produces ammonium ions to promote acid excretion. Therefore, when HUA occurs and blood uric acid is excessive, the kidneys will take up more glutamine and glutamate to compensate for the excess uric acid.

Glutamic acid can undergo interconversion with arginine, ornithine, and proline through the urea cycle in living organisms. Meanwhile, high levels of uric acid can accelerate arginine metabolism by upregulating ornithine decarboxylase, thereby affecting the body to varying degrees [29]. Other studies have shown that [30] serum ornithine levels in gout patients are significantly elevated statistically. Therefore, the changes in ornithine in the body can to some extent affect and regulate the content of uric acid.

In this study, compared with the blank control group, the HUA model group showed an upregulation of arginine concentration in the blood, which may be a spontaneous protective response in the human body helping to alleviate the pathological process of HUA. And glutamate showed a decreasing trend, indicating that feeding HUA rats with modeling-specific feed may accelerate the uptake of glutamate by the rat liver and produce a large amount of uric acid. After preventive treatment with ELITEA compound tea, the concentration of glutamate showed a downward trend, indicating that ELITEA compound tea may have played a role in preventing HUA by affecting the liver’s absorption and metabolism of glutamate. The increase in the ornithine content in HUA rats also indicates that their bodies spontaneously produce protective mechanisms, promoting the rise of ornithine to affect other circulatory modes of the body, thereby regulating uric acid.

## 5. Conclusions

This study has demonstrated that ELITEA compound tea can effectively reduce the serum uric acid levels in HUA model rats. Based on an in-depth analysis of untargeted metabolomics, ELITEA compound tea mainly regulates the arginine biosynthesis pathway by modulating three important metabolites, arginine, glutamate, and ornithine, to reduce serum uric acid.

## Figures and Tables

**Table 1:** OPLS-DA model evaluation parameters.

Sample Comparison Group	R2X (cum) ^1^	R2Y (cum) ^2^	Q2 (cum) ^3^	RMSEE ^4^
A.vs.B	0.414	0.998	0.913	0.0265
B.vs.D	0.459	0.996	0.926	0.0361
A_B_D ^5^	0.26	0.989	0.763	0.112

^1^ R2X: The explanatory power of the model on the X matrix; ^2^ R2Y: The explanatory power of the model on the Y matrix; ^3^ Q2: Indicates the predictive ability of the model; ^4^ RMSEE: Root mean square error of estimation; ^5^ A: Blank control group; B: HUA model group; D: ELITEA compound tea prevention experimental group.

**Table 2:** Some significant differential metabolites among groups A, B, and D.

Metabolite Name	Formula	RT (min)	Reference *m*/*z*	HMBD ID	A vs. B			B vs. D		
					Trend	*p*.Value	VIP	Trend	*p*.Value	VIP
Biotin	C_10_H_16_N_2_O_3_S	5.028	267.07742	HMDB0000030	↓	<0.001	1.85	↑	<0.001	1.85
3-(6,7-Dimethoxy-1,2,3,4-tetrahydroisoquinolin-1-yl)propan-1-ol	C_14_H_21_NO_3_	6.278	252.15938	---	↓	<0.001	1.83	↑	<0.001	1.84
2-hydroxy-4-methoxy-6-methylbenzoic acid	C_9_H_10_O_4_	5.121	181.10002	---	↓	<0.001	1.84	↑	<0.001	1.84
Kobusone	C_14_H_22_O_2_	6.046	261.12515	HMDB0036790	↓	<0.001	1.84	↑	<0.001	1.84
6-Hydroxypseudooxynicotine	C_10_H_14_N_2_O_2_	5.808	195.11278	HMDB0240264	↓	<0.001	1.84	↑	<0.001	1.83
6-n-Propyluracil	C_7_H_10_N_2_O_2_	4.858	155.0815	HMDB0247095	↓	<0.001	1.84	↑	<0.001	1.83
Ncgc00380138-01!	C_11_H_15_NO_3_	6.182	227.13902	---	↓	<0.001	1.85	↑	<0.001	1.83
Anisole	C_7_H8_O_	7.52	107.04969	HMDB0033895	↓	<0.001	1.84	↑	<0.001	1.83
Hydroxytoluic acid	C_8_H_8_O_3_	7.518	151.04002	HMDB0002390	↓	<0.001	1.84	↑	<0.001	1.83
Albuterol	C_13_H_21_NO_3_	8.675	240.15938	HMDB0001937	↓	<0.001	1.85	↑	<0.001	1.83
1,3,6,7-tetrahydroxy-8-(3-hydroxy-3-methylbutyl)-2-(3-methylbut-2-en-1-yl)-9H-xanthen-9-one	C_23_H_26_O_7_	5.127	437.15701	HMDB0029511	↓	<0.001	1.83	↑	<0.001	1.82
2-{2-benzimidazol-2-yl-1-[(4-methylphenyl)methyl]ethyl}benzimidazole	C24H22N4	5.841	401.15381	---	↓	<0.001	1.84	↑	<0.001	1.82
3′,5′-Dideoxythymidine	C_10_H_14_N_2_O_3_	5.028	211.10771	HMDB0246113	↓	<0.001	1.83	↑	<0.001	1.82
L-Acetylleucine	C_8_H_15_NO_3_	5.098	196.09442	HMDB0011756	↓	<0.001	1.82	↑	<0.001	1.82
3-carboxy-4-methyl-5-pentyl-2-furanpropanoic acid	C_14_H_20_O_5_	8.413	267.12326	HMDB0061643	↓	<0.001	1.81	↑	<0.001	1.82
Valaciclovir	C_13_H_20_N_6_O_4_	8.372	323.14731	HMDB0014716	↓	<0.001	1.81	↑	<0.001	1.81
PyroGlu-Pro	C_10_H_14_N_2_O_4_	5.124	227.10258	---	↓	<0.001	1.81	↑	<0.001	1.81
[2-(5-fluoro-2-methylindol-3-yl)ethyl][(2-methoxy-4,5-dimethylphenyl)sulfonyl] amine	C_20_H_23_FN_2_O_3_S	6.078	435.14001	---	↓	<0.001	1.85	↑	<0.001	1.81
L,L-Cyclo(prolylalanyl)	C_8_H_12_N_2_O_2_	5.06	169.09715	HMDB0303321	↓	<0.001	1.81	↑	<0.001	1.81
Alanylnorleucine	C_9_H_18_N_2_O_3_	5.814	185.129	---	↓	<0.001	1.81	↑	<0.001	1.81
Sinapoylhexoside	C_17_H_22_O_10_	5.793	385.11401	---	↑	<0.001	1.84	↓	<0.001	1.83
3,5-Di-tert-butyl-4-hydroxybenzoic acid	C_15_H_22_O_3_	9.404	251.16418	HMDB0240642	↑	<0.001	1.80	↓	<0.001	1.80
cis-15-Octadecenoic acid	C18H34O2	10.819	283.26315	HMDB0304765	↑	<0.01	1.48	↓	<0.001	1.79
Octadeca-9,12-dienal	C_18_H_32_O	10.812	265.25259	HMDB0247717	↑	<0.01	1.54	↓	<0.001	1.79
Cadabicilone	C_15_H_22_O_3_	9.391	249.14906	HMDB0037559	↑	< 0.001	1.81	↓	<0.001	1.79
3,5-Dihydroxycinnamic acid sulfate	C_9_H_8_O_7_S	5.552	258.99124	HMDB0240449	↑	<0.001	1.76	↓	<0.001	1.77
L-Monomenthyl glutarate	C_15_H_26_O_4_	8.79	269.17584	HMDB0303264	↑	<0.001	1.68	↓	<0.001	1.76
Dichlorprop	C_9_H_8_C_l2_O_3_	5.086	232.97772	HMDB0251202	↑	<0.001	1.76	↓	<0.001	1.76
Benzoic acid + 2O, O-Hex	C_13_H_16_O_9_	4.945	315.07211	---	↑	<0.01	1.53	↓	<0.001	1.75
(R)-3-Hydroxy-Octadecanoic acid	C_18_H_36_O_3_	10.802	299.25861	HMDB0010737	↑	<0.01	1.49	↓	<0.001	1.75
Phellopterin	C_17_H_16_O_5_	4.941	339.06973	HMDB0256386	↑	<0.01	1.47	↓	< 0.001	1.75
7,4′-dihydroxy-3′-(gamma,gamma-dimethylallyl)isoflavone	C_20_H_18_O_4_	9.271	321.20209	HMDB0255518	↑	<0.01	1.56	↓	<0.001	1.73
N-[1-(4-methoxy-6-oxopyran-2-yl)-2-methylpropyl]acetamide	C_12_H_17_NO_4_	0.911	257.14951	---	↑	<0.05	1.30	↓	<0.001	1.72
3-(3,5-dihydroxyphenyl)-1-propanoic acid sulphate	C_9_H_10_O_7_S	5.404	261.0069	HMDB0061117	↑	< 0.001	1.79	↓	<0.001	1.71
Korseveriline	C_27_H_45_NO_3_	9.506	454.32913	---	↑	<0.001	1.80	↓	<0.001	1.70
Racivir	C_8_H_10_FN_3_O_3_S	5.94	246.0354	HMDB0015017	↑	<0.001	1.35	↓	<0.001	1.70
beta-Gentiobiose	C_12_H_22_O_11_	5.165	381.07935	HMDB0248216	↑	<0.001	1.83	↓	<0.001	1.69
1-(5,7-Dihydroxy-2,2,6-trimethyl-2H-1-benzopyran-8-yl)-3-phenyl-2-propen-1-one	C_21_H_20_O_4_	7.25	335.12881	---	↑	<0.001	1.75	↓	<0.001	1.67
15,16-DiHODE	C_18_H_32_O_4_	8.981	311.22223	HMDB0010208	↑	<0.01	1.47	↓	<0.001	1.65
8-Hydroxy-9,10-epoxystearic acid	C_18_H_34_O_4_	9.312	337.23492	---	↑	<0.01	1.53	↓	<0.001	1.65

## Data Availability

The original contributions presented in this study are included in the article. Further inquiries can be directed to the corresponding author.
